# FMAlign2: a novel fast multiple nucleotide sequence alignment method for ultralong datasets

**DOI:** 10.1093/bioinformatics/btae014

**Published:** 2024-01-10

**Authors:** Pinglu Zhang, Huan Liu, Yanming Wei, Yixiao Zhai, Qinzhong Tian, Quan Zou

**Affiliations:** Institute of Fundamental and Frontier Sciences, University of Electronic Science and Technology of China, Chengdu 610054, Sichuan, China; Yangtze Delta Region Institute (Quzhou), University of Electronic Science and Technology of China, Quzhou 324003, Zhejiang, China; School of Computer Science and Technology, Southwest University of Science and Technology, Mianyang 621010, Sichuan, China; School of Computer Science and Technology, Xidian University, Xi’an 710071, Shaanxi, China; Institute of Fundamental and Frontier Sciences, University of Electronic Science and Technology of China, Chengdu 610054, Sichuan, China; Yangtze Delta Region Institute (Quzhou), University of Electronic Science and Technology of China, Quzhou 324003, Zhejiang, China; Institute of Fundamental and Frontier Sciences, University of Electronic Science and Technology of China, Chengdu 610054, Sichuan, China; Yangtze Delta Region Institute (Quzhou), University of Electronic Science and Technology of China, Quzhou 324003, Zhejiang, China; Institute of Fundamental and Frontier Sciences, University of Electronic Science and Technology of China, Chengdu 610054, Sichuan, China; Yangtze Delta Region Institute (Quzhou), University of Electronic Science and Technology of China, Quzhou 324003, Zhejiang, China

## Abstract

**Motivation:**

In bioinformatics, multiple sequence alignment (MSA) is a crucial task. However, conventional methods often struggle with aligning ultralong sequences. To address this issue, researchers have designed MSA methods rooted in a vertical division strategy, which segments sequence data for parallel alignment. A prime example of this approach is FMAlign, which utilizes the FM-index to extract common seeds and segment the sequences accordingly.

**Results:**

FMAlign2 leverages the suffix array to identify maximal exact matches, redefining the approach of FMAlign from searching for global chains to partial chains. By using a vertical division strategy, large-scale problem is deconstructed into manageable tasks, enabling parallel execution of subMSA. Furthermore, sequence-profile alignment and refinement are incorporated to concatenate subsets, yielding the final result seamlessly. Compared to FMAlign, FMAlign2 markedly augments the segmentation of sequences and significantly reduces the time while maintaining accuracy, especially on ultralong datasets. Importantly, FMAlign2 enhances existing MSA methods by conferring the capability to handle sequences reaching billions in length within an acceptable time frame.

**Availability and implementation:**

Source code and datasets are available at https://github.com/malabz/FMAlign2 and https://zenodo.org/records/10435770.

## 1 Introduction

Multiple sequence alignment (MSA) plays a crucial role in bioinformatics, particularly in analyzing biological sequences. As noted in a Nature publication (Van Noorden *et al.* 2014), MSA remains one of the most fundamental modeling methodologies within the biological sciences. It serves as a foundational tool in a broad range of computational analyses, including but not limited to domain analysis, phylogenetic reconstruction, and motif identification. The accuracy of sequence alignment is vital as it critically impacts the validity and reliability of subsequent analyses. Nevertheless, with the ongoing expansion of biological sequence scales, the limitations of numerous extant MSA approaches in managing ultralong sequences are becoming increasingly evident (Lewin *et al.* 2018). Even though most current methods for MSA are built to align a large number of sequences, they struggle when aligning long sequences because of the higher computing costs related to sequence length (Zhang *et al.* 2022). To this end, our primary goal is to improve the performance of alignment methods, especially focusing on the alignment of multiple ultralong sequences.

Conventional methods often fall short when tackling ultralong sequences, increasing the adoption of acceleration techniques. Among these techniques, the vertical division strategy stands out. This strategy seeks common segments/minimizers to divide all the sequences and aligns every generated sub-sequence in parallel using the existing MSA method, enabling MSA methods to handle ultralong sequences more effectively. FAME (Naznooshsadat *et al.* 2020), a novel vertical-division-based method for aligning similar sequences, has gained attention in recent years. This method utilizes hash tables to detect k-mers and minimizers, potentially incorporating nonoverlapping anchors into a single chain. However, this proves inefficient for long, similar sequences. To address these limitations, FMAlign (Liu *et al.* 2022) was developed, which prioritizes common segments as candidate anchors. FMAlign uses the FM-index (Hon *et al.* 2004), a widely accepted full-text index, to efficiently query common segments of varying lengths, speeding up anchor searches across multiple sequences and enhancing the alignment process. Both FAME and FMAlign search for common seeds across all sequences, forming global chains to segment the sequences. When dealing with ultralong sequences or sequences with low similarity, these two methods struggle to find a sufficient number of global chains for acceleration.

To overcome the limitations of FAME and FMAlign, FMAlign2 utilizes Maximal Exact Matches (MEMs) instead of k-mers to identify partial chains in sequences. Although methods like MUMmer (Marçais *et al.* 2018) also partition sequences using MUM (Maximal Unique Match) or MEMs for pairwise sequence alignment, there are currently few methods that use MEMs to segment multiple sequences. It constructs suffix array (Manber and Myers 1993) and longest common prefix (LCP) array, identifies maximal exact matches (MEMs), and generates a colinear set of MEMs for alignment. FMAlign2 uses the striped Smith–Waterman (SSW) (Zhao *et al.* 2013) algorithm to identify similar substrings for each MEMs in sequences where MEMs are absent. The identified similar substrings, combined with MEMs, form the partial chains used for subsequent sequence segmentation to generate segments. External tools such as MAFFT (Katoh *et al.* 2002) and HAlign (Zou *et al.* 2015, Tang *et al.* 2022) align these segments in parallel. FMAlign2 leverages sequence-profile alignment based on FFT/K-Band (Wei *et al.* 2022) to incorporate fragments into the backbone. Finally, FMAlign2 concatenates and refines these segments to generate the final result.

## 2 Materials and methods

### 2.1 Overview of FMAlign2

**Figure 1.**
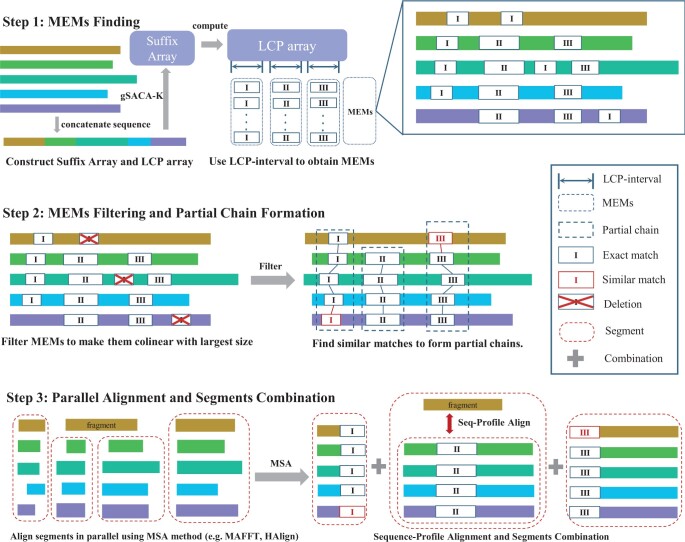
Workflow of FMAlign2: In Step 1, FMAlign2 constructs suffix array for the string collections, along with the LCP array. By traversing the LCP array, LCP-intervals are obtained, which lead to the identification of MEMs. In Step 2, FMAlign2 filters MEMs to ensure their colinearity with the largest size. Subsequently, local alignments are performed to detect similar matches, which are then appended to the existing MEMs, resulting in the formation of partial chains. In Step 3, sequences are divided into segments by the partial chains. Leveraging parallel processing, FMAlign2 aligns these segments using MSA methods like MAFFT and HAlign. Then fragments are aligned to segments through sequence-profile alignment, allowing the segments to be assembled into the ultimate MSA result.

We present FMAlign2, an innovative MEMs-based approach for MSA. The methodology unfolds in three primary steps (Fig. 1):

- **Step 1: MEMs Finding—**In the preprocessing stage, nucleotides that fall outside the defined character set Σ={A,C,G,T} are replaced with the gap “-”. Following this, sequences are concatenated into a single sequence. The gSACA-K algorithm (Louza *et al.* 2020) is then used to establish this concatenated sequence’s suffix array and LCP array. Finally, MEMs are generated through the traversal of the LCP array and left-extension of LCP-interval.
- **Step 2: MEMs Filtering and Partial Chain Formation—**FMAlign2 filters the MEMs to ensure their colinearity and gives precedence to those with the largest size. It then conducts the Striped Smith–Waterman (SSW) algorithm to identify similar matches, which are subsequently appended to the existing MEMs, culminating in the formation of partial chains.
- **Step 3: Parallel Alignment and Segments Combination—**The sequence data are partitioned into discrete segments. Parallel alignment is then executed using traditional MSA methods, such as HAlign and MAFFT. Subsequently, Sequence-Profile alignment based on FFT/K-Band is deployed to incorporate the fragments into the backbone. Finally, aligned segments are concatenated, with refinement applied to yield the final result.

### 2.2 MEMs finding

One of the core concepts in FMAlign2 is MEMs. Thus, it’s essential to define the MEMs finding problem and explain the method to obtain MEMs by constructing a suffix array and extending LCP intervals.

MEMs finding definition—In pairwise alignment, MEMs are exact matches between two sequences that cannot be extended to the left or right without introducing a mismatch (Vyverman *et al.* 2013). These matches are widely used as seeds for pairwise sequence alignment tools, such as MUMmer (Marçais *et al.* 2018). In the context of multiple sequences, the definition of MEMs expands as follows: Suppose we have *n* sequences, and let *S* be the concatenated sequence of these *n* sequences, distinguishing each string with unique separator symbols not found in any string and smaller than any symbol in the alphabet. Then, a MEMs within *S* can be represented using a (k+1)-tuple: $\left(l,p_{1},p_{2},\ldots ,p_{k}\right)$, where *l* denotes the length of the substrings, *k* denotes the number of substrings, and $p_{i}\left(1\leq i\leq k\right)$ is the starting position of the *i*th substring. The MEMs finding problem definition is: given a concatenated sequence *S* and an integer $l_{\min}$, find all MEMs of length at least $l_{\min}$ in *S*.

Suffix array and LCP array—The construction of the suffix array, as detailed by Manber and Myers (1993), forms a critical foundation in bioinformatics. This is attributable to the capacity of suffix arrays, paired with additional data structures (Muthukrishnan 2002), to effectively tackle string-related problems. Manber and Myers (1993) defined the suffix array of a string as the array of the indexes of the lexicographically sorted suffix strings. On the other hand, the LCP array provides the length of the shared prefix between two suffixes in the SA. FMAlign2 uses the gSACA-K (Louza *et al.* 2020) method to construct suffix array and LCP array of the concatenated string *S* with O(N) time complexity.

LCP interval finding—The LCP-interval (Abouelhoda *et al.* 2002) is an interval[*i*..*j*] of lcp-value $l_{\min}$ in the LCP array, which satisfies the following properties:

1.
$\text{LCP}[i]<l_{\min}$ ,
2.
$\text{LCP}[k]\geq l_{\min}$ for all *k* with i+1≤k≤j,
3.
$\text{LCP}[k]=l_{\min}$ for at least one *k* with i+1≤k≤j,
4.
$\text{LCP}[j+1]<l_{\min}$ .

An LCP-interval is a subarray of LCP where all values are at least $l_{\min}$, and at least one value equals $l_{\min}$. The LCP values just before and after this section are always less than $l_{\min}$. In FMAlign2, we use a two-pointer approach to traverse the LCP array in search of LCP-intervals, with the pseudocode provided in Supplementary Algorithm S1. Combined with the suffix array, finding all the LCP intervals means identifying all positions of common substrings of length $l_{\min}$ that appear at least twice. The LCP interval ensures that the characters to the right of these common substrings are not the same, but it does not guarantee this for the characters on the left.

Extending LCP interval to MEMs—According to the definition of the MEMs finding problem, to acquire MEMs of at least length $l_{\min}$, one simply extends the identified LCP intervals to the left. As shown in Supplementary Fig. S1, the left extension involves an iterative process of comparing nucleotides to the left of the common substrings across all sequences until a mismatch occurs or a sequence boundary is reached. Consequently, by extending the LCP-intervals in this manner, we can systematically pinpoint all MEMs across the sequences involved.

### 2.3 MEMs filtering and partial chain formation

Although FMAlign2 uses MEMs and MUMmer (Marçais *et al.* 2018) uses MUM to segment pair sequences in a manner that seems quite similar, segmenting multiple sequences is a more complex problem than segmenting just two sequences. Considering the added challenge of segmenting multiple sequences, it’s vital to emphasize the role of MEMs colinearity and size. If two MEMs each contain at most one substring per sequence and these substrings consistently maintain the same relative order across all sequences without overlap, we refer to these two MEMs as being colinear. Two MEMs being colinear means they will not conflict when segmenting sequences. The size of the *i*th MEMs is defined as the product of its substring length $l_{i}$ and the number of its substrings $k_{i}$. We also establish a minimum sequence coverage threshold c(0≤c≤1). MEMs that do not cover a proportion greater than *c* of the total sequences *n* will be discarded. Given that larger MEMs are more likely to contribute to MSA, our goal is to select a set of MEMs that not only have the largest size and are colinear with each other, but also possess a substring count exceeding the floor value of c×n. We perform preprocessing and use either global or local dynamic programming modes to filter MEMs; specific details can be found in the Supplementary Section S4.

Chains formation—While these filtered MEMs represent exact matches within these subsets, variations, insertions, and deletions can cause potential similar regions to remain undetected. Consequently, FMAlign2 uses the striped Smith–Waterman (SSW) (Zhao *et al.* 2013) algorithm to identify similar substrings for each valid MEMs in sequences where MEMs are absent. The SSW algorithm, accelerated by Simple Instruction Multiple Data (SIMD), allows rapid local alignment. It aligns the exact match in MEMs against the corresponding regions between the two chains. If the proportion of gaps in the local alignment results exceeds a predefined threshold (default value is 0.8), the identified similar substring will be discarded because of low quality. The identified similar substrings, combined with MEMs, form the partial chains used for subsequent sequence segmentation.

### 2.4 Parallel alignment and segments combination

Unlike FAME (Naznooshsadat *et al.* 2020) and FMAlign (Liu *et al.* 2022), which use global chains, FMAlign2 segments sequences utilizing partial chains that appear in a subset of sequences. A global chain refers to a chain that exists in all sequences, with its substrings being completely identical across all sequences. On the other hand, a local chain may only appear in some sequences, with its substrings being similar but not necessarily identical. As illustrated in Step 3 of Fig. 1, We define the collection of substrings resulting from the segmentation by a partial chain as a segment, while the remaining individual substring is referred to as fragment.

Parallel alignment—FMAlign2 integrates with MAFFT (Katoh *et al.* 2002), HAlign2 (Zou *et al.* 2015), and HAlign3 (Tang *et al.* 2022) for aligning segments in parallel. While these tools were chosen mainly for benchmarking against FMAlign, it’s worth noting that with the appropriate setup, FMAlign2 can collaborate with most MSA software. If a particular segment remains too vast for alignment, FMAlign2 recursively applies itself to the set with a reduced MEMs minimum length parameter. This recursion is limited to two iterations.

Sequence-profile alignment—After the segments were aligned, we incorporated fragments that the SSW algorithm could not previously match, to the backbone. As shown in Supplementary Fig. S3, FMAlign2 initially calculates the length of unaligned fragments within each sequence, and orders them from shortest to longest. Priority is given to aligning sequences that possess shorter unaligned fragments. During each sequence-profile alignment, we identify the partial chains flanking the fragment. Segments and partial chains previously aligned between these chains are merged into a new aligned set, to which the associated segment is then incorporated into this new set. FMAlign2 uses an improved sequence-profile based on the FFT/K-Band strategy proposed by Wei *et al.* (2022). This method leverages the fast Fourier transform (FFT) to identify homologous segments and uses the K-Band to minimize the dynamic programming matrix. Ultimately, all fragments are aligned to the backbone, leaving only the subsequence set that encompasses all sequences.

Segments combination and refinement—When forming partial chains, errors in local alignment might incorrectly allocate base pairs, which should belong to the edges of the local chain, into adjacent segments. To refine these, we inspect and quantify the gaps at each merging point as we concatenate the segments. A specific example and the steps of refinement are shown in Supplementary Fig. S4. The final optimized MSA result is obtained through refinement during the concatenation of all segments.

## 3 Results and discussion

FMAlign2 supports parallel acceleration on both Linux and Windows operating systems, utilizing OpenMP on Windows and the pthread library on Linux. The experiments were run with Ubuntu 20.04.4 LTS, an Intel(R) Xeon(R) Gold 6230 CPU @ 2.10 GHz, 80 CPUs, and approximately 1 TB of memory. All methods, including MAFFT, HAlign2, HAlign3, FMAlign, and FMAlign2, used 80 threads for execution. For brevity, when MAFFT, HAlign2, and HAlign3 are combined with FMAlign or FMAlign2, we refer to them as M, H2, and H3, respectively. To ensure an equitable comparison, we standardized the settings across all MSA methods. These settings were fine-tuned to maximize both speed and accuracy.

Q (quality) score (Edgar 2004) is the number of correctly aligned residue pairs divided by the number of residue pairs in the reference alignment. Total column(TC) score (Edgar 2004) is the number of correctly aligned columns divided by the number of columns in the reference alignment. For simulated datasets, We use the Q and TC scores calculated by the MUSCLE Q-Score (Edgar 2004) to evaluate the alignment accuracy with reference alignment to compare. It’s noteworthy that the Q-score program fail to produce Q score and TC scores when faced with incorrect alignments. For real datasets, we choose the average sum-of-pairs (SP) score value, equal to the sum of every pairwise alignment score divided by the number of sequences. In our SP scoring system, matches scored 0, mismatches 1, and gaps 2 according to Liu *et al.* (2022). This implies that a lower SP score corresponds to higher alignment quality.

**Figure 2.**
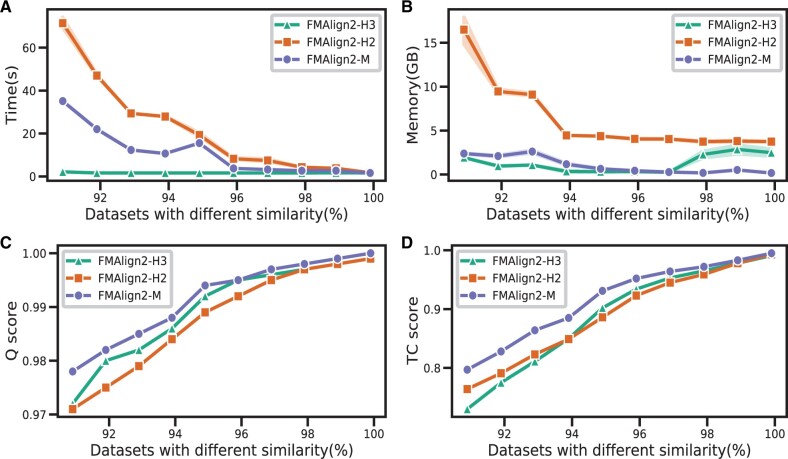
Comparison of three methods in FMAlign2 on hierarchical tree simulated different similarity datasets. (A) Comparison of the three methods on time. (B) Comparison of the three methods on memory. (C) Comparison of the three methods on Q score. (D) Comparison of the three methods on TC score.

As shown in Fig. 2, to explore the relationship between sequence similarity and performance in terms of runtime, memory usage, and alignment quality, a mitochondrial-like dataset comprising 100 sequences with different similarities ranging from 90% to 99% is simulated using INDELible v1.03 (Fletcher and Yang 2009) provided by Tang *et al.* (2022). As demonstrated in Fig. 2A and B, a rise in sequence similarity corresponds with a gradual decline in computational time and memory consumption across all three methods. We also observe that the curves for runtime and memory usage are not smooth. For instance, at a 93% similarity level, there’s an anomalous increase in memory consumption. This suggests that FMAlign2 exhibits instability of segmentation under the same parameter settings. It is particularly noteworthy that FMAlign2-H3 consumes significantly less time than FMAlign2-M and FMAlign2-H2. The memory requirements of FMAlign2-H2 dramatically surpass those of the other two methods. Regarding alignment quality, the alignment quality achieved by FMAlign2-M consistently surpasses that of the other two methods. As such, while FMAlign2-H3 demonstrates exceptional speed in alignment, FMAlign2-M notably outperforms its counterparts in alignment quality.

**Figure 3.**
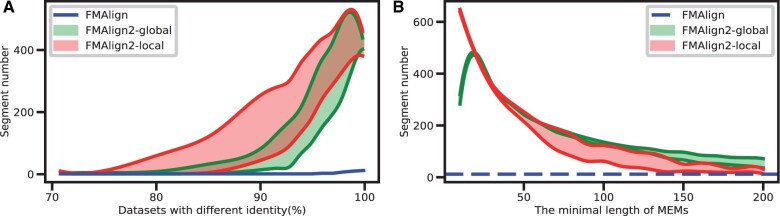
Comparison of two modes in FMAlign2 and FMAlign for segment number. The lower curve represents a sequence coverage of 0.8, while the upper curve corresponds to a sequence coverage of 0.4. (A) On different simulated sequence similarity datasets, the comparison of the sequence segmentation numbers of the local, global mode in FMAlign2 and FMAlign. (B) As the minimum length of FMAlign2’s MEMs changes, the comparison of the number of segments between the two modes of FMAlign2 and FMAlign.

In methods where vertical division strategy serves as the core concept, the number of segments made on the dataset is of utmost concern. Optimal determination of the quantity of segments can notably enhance the efficiency of the alignment. If the number of divisions is too small, each subsequence becomes too long, wasting resources and reducing efficiency. Hence, this experiment compares the global and local modes of FMAlign2, as well as FMAlign, for the segment number. For FMAlign2’s two modes, we tested different sequence coverage *c* of MEMs, ranging from 0.4 to 0.8. Sequence coverage denotes the least count of substrings derived from the filtered MEMs, with the lower the value of *c*, the higher the quantity of MEMs detected. In Fig. 3, the lower curve represents a coverage of 0.8, while the upper curve corresponds to a coverage of 0.4. To ensure a fair comparison between FMAlign2 and FMAlign, we set the minimum length of MEMs to match the k-mer size of *k* = 39, as used in FMAlign. As shown in Fig. 3A, for the same mitochondrial-like dataset with different similarities ranging from 70% to 99%, we observe a corresponding escalation in the number of segments within the three evaluated methods, concurrent with an increase in dataset similarity. We observe that when sequence similarity approaches either 100% or 70%, the segment count in both modes tends to converge. In most instances, the local mode has a higher segment count than the global mode, indicating its better suitability for handling low-similarity scenarios. Although FMAlign2’s modes typically have a significantly higher segment count than FMAlign, both methods face challenges in segmenting sequences when similarities are exceptionally low. We also explore the impact of the minimum MEMs length parameter, *l*, on segment count using the Human Mitochondrial Genome (mt) Dataset (Ingman and Gyllensten 2006). Figure 3B shows that when *l* is very small, FMAlign2’s local mode produces a notably higher number of segments than its global mode. This surge is attributed to the abundance of MEMs when *l* is minimal, leading to an increased frequency of overlaps among them. While the global mode dismisses many MEMs in its approach of using full MEMs for dynamic programming, the local mode preserves the entire MEMs by only needing partial substring deletion. Hence, when facing numerous overlaps, the local mode can segment sequences more effectively. It’s also observed that as *l* increases, the overlap among MEMs decreases, and the global mode starts producing more segments than the local mode. Nevertheless, FMAlign2’s segment counts in both modes substantially exceed that of FMAlign, highlighting that shifting from global to partial chain search can considerably increase sequence segments. Both Fig. 3A and B indicate that in complex scenarios, like low sequence similarity or frequent MEMs overlaps, the local mode outshines the global mode. However, for simpler cases, the global mode is often the superior choice.

For FMAlign2, its notable advantage is handling ultralong sequences that are challenging for other methods. Consequently, we selected two human Y chromosomes and extracted different lengths from these chromosomes, truncating them at various points ranging from 10 000 bp to 15Mbp. We divided eight methods into three groups for comparison according to their core methodologies. As illustrated in the Supplementary Fig. S5, for methods centered on HAlign2, both HAlign2 and FMAlign-H2 cease to function correctly once sequence length surpasses 100 000. When the length reaches 500 000, FMAlign-H2 also becomes inoperable. In contrast, within the experiments utilizing MAFFT as the core method, MAFFT can process sequences over 5 million in length. We note that when the sequence is truncated to a length of 5 million, there’s a marked surge in the runtime. This can be attributed to FMAlign2’s instability of segmentation. Based on our observations in the experiment, the segmentation of this sequence produced an extremely long segment, and aligning this segment resulted in the unusual spike in execution time. The experimental results demonstrate that vertical division technology offers a significant advantage when aligning ultralong sequences. FMAlign can handle sequences on the order of millions, while FMAlign2 can provide results within an acceptable timeframe even when faced with sequences 10 Mbp long or longer.

To test FMAlign2’s performance on real datasets, we choose the long and similar datasets to serve as our benchmark. This dataset provided by Naznooshsadat *et al.* (2020) includes five sequence sets of *Variola* (*VARV*), *Mycoplasma genitalium* (*M.genitalium*), *Mycoplasma bovis* (*M.bovis*), *Streptococcus pneumoniae* (*S.pneumoniae*), and *Escherichia coli* (*E.coli*). Each set contains an equal number of sequences but differs in average lengths, allowing us to assess the performance of the methods concerning the sequence length. Detailed dataset information is provided in Supplementary Table S1. Table 1 demonstrates the performance of different methods across five datasets. HAlign2 on its own was unable to complete alignments for any dataset. FMAlign-H2 managed to align three out of the five datasets, while FMAlign2-H2 achieved successful alignment in all cases, underscoring FMAlign2’s enhanced robustness. When comparing runtime and average SP scores across these datasets, both FMAlign and FMAlign2 not only reduced alignment time but also preserved the original quality of alignments (except for *E.coli*), with FMAlign2 exhibiting a more pronounced reduction in time. Notably, the combination of HAlign3 and FMAlign2 not only preserved but in some cases even significantly enhanced the quality of the alignment.

**Table 1.** Time consumption and average SP scores for virus genome datasets.^a^

Method	*VARV*		*M.genitalium*		*M.bovis*		*S.pneumonia*		*E.coli*	
	Time	Average SP	Time	Average SP	Time	Average SP	Time	Average SP	Time	Average SP
MAFFT	19.14 s	2705.66	3 min 28 s	6637	4 min 1 s	7149	16 min 10 s	1 342 439.83	28 min 8 s	1 647 099.66
HAlign2	—	—	—	—	—	—	—	—	—	—
HAlign3	1.06 s	4298.16	1.06 s	10 122.33	1.07 s	10 599.16	—	—	—	—
FMAlign-M	0.98 s	2866.66	2.67 s	6823	2.96 s	7280.5	57.33 s	888 375.33	11 h 49 min 19 s	2 808 974
FMAlign-H2	1.69 s	2083.83	2.97 s	6686.66	3.55 s	7385	—	—	—	—
FMAlign2-M	0.23 s	2701.66	0.56 s	6843.33	0.87 s	7172	52.64 s	887 156.33	7 min 52 s	1 518 278.16
FMAlign2-H2	0.44 s	2753	1.39 s	7545.33	1.77 s	8680.5	23.55 s	872 073.5	34.57 s	1 589 134.5
FMAlign2-H3	0.52 s	2785.83	0.95 s	7313	0.86 s	8029.66	9.96 s	952 443.83	15.55 s	1 543 641.33

a “—” indicates that the method cannot complete the alignment on the dataset due to memory overflow.

To evaluate our method’s performance on large-scale datasets, we also utilize the mtDB benchmark dataset (Ingman and Gyllensten 2006), duplicating the genomes from this dataset 20, 50, and 100 times. Detailed dataset information is provided in Supplementary Table S1. As Supplementary Table S2 in supplement material shows, comparative experiments on eight methods using the mtDB indicate that FMAlign, when combined with MAFFT and HAlign2, can reduce alignment time while preserving accuracy. However, this time-saving advantage decreases as the number of sequences increases. When handling mt (100×), FMAlign’s performance converges with that of the stand-alone methods. In contrast, FMAlign2 demonstrates a more pronounced acceleration, maintaining over 50% time reduction compared to the original methods even on the mt (100×). Notably, HAlign3’s unique mechanism affords it the fastest alignment speed on the mtDB dataset among the tested methods. Yet, for high-count sequence datasets like mtDB, using FMAlign2-H3 results in longer alignment times than HAlign3 alone. In such cases, the process of constructing suffix arrays by FMAlign2 appears to be a detriment to the efficiency of HAlign3.

We also document the peak memory usage of different methods. As shown in Supplementary Tables S3 and S4, it is observed that the peak memory usage when running FMAlign2 is not stable. For large datasets, the peak memory usage of FMAlign2 and FMAlign exceeds that of the combined methods. This is attributed to the fact that the peak memory consumption for both FMAlign2 and FMAlign occurs during the parallel alignment phase, where the cumulative memory usage of multiple tasks running concurrently may spike momentarily. Conversely, for smaller datasets, due to the rapid completion of subtasks, there will not be a large number of tasks running in parallel, thus the peak memory usage of FMAlign and FMAlign2 will be less than that of the combined methods. Lastly, owing to the uncertainty of the segmentation process, it is difficult to compare the peak memory usage between FMAlign2 and FMAlign.

## 4 Conclusion

In this paper, we propose a novel method called FMAlign2 for ultralong sequences based on MEMs. Unlike FAME and FMAlign, which relies on global chain segmenting sequences, FMAlign2 adopts partial chain strategy, augmenting the segment quantity across datasets of various similarity levels. FMAlign2 also applies a vertical division strategy, deconstructing large-scale problem into manageable subtasks. Moreover, the method incorporates sequence-profile alignment and refinement strategies to concatenate these segments and generate the final result. FMAlign2 demonstrates significant advantages over FMAlign, particularly in terms of sequence segmentation. It significantly reduces time consumption while maintaining the accuracy of the alignment. The introduction of FMAlign2 provides a powerful solution for the alignment of ultralong sequences and presents a new perspective for dealing with large-scale sequence data alignment in the future. However, FMAlign2 has certain limitations when confronted with low similarity and extremely large datasets. When faced with such conditions, the alignment time would significantly increase, and it may even fail to finish the alignment. Our future work will explore a combination of horizontal and vertical division strategy to overcome these limitations.

## Supplementary Material

btae014_Supplementary_Data
