# Supplementary material for: FMAlign2: a novel fast multiple nucleotide sequence alignment method for ultralong datasets
Source: Bioinformatics. 2024 Jan 10;40(1):btae014. doi: 10.1093/bioinformatics/btae014 (PMC10809904; doi:10.1093/bioinformatics/btae014)
Supplement: btae014_Supplementary_Data [file btae014_supplementary_data.pdf]

Supplementary Material

November 11, 2023

1 Suffix Array and LCP Array Construction

The construction of the suffix array, as outlined by Manber and Myers [1993], serves as a fundamental component in bioinformatics and various applied string processing fields. This is attributable to the capacity of suffix arrays, in conjunction with additional data structures, to effectively tackle string-related problems such as pattern matching. Commonly employed additonal data structures include the Longest Common Prefix array (LCP) and the Document Array (DA) [Muthukrishnan, 2002]. Despite the existence of numerous efficient algorithms for creating suffix arrays of individual strings and associated additional data structures, a scarcity of algorithms focusing on constructing suffix array for string collections remains. Currently, gsufsort [Louza et al., 2020] stands as the most expeditious algorithm for generating suffix array of string collections along with additional data structures. As an open-source software, gsufsort supports fasta, fastq, and text files containing multiple strings as input, subsequently constructing suffix array and additional data structures before writing them to disk. FMAlign2 utilizes gsufsort to address suffix array and additonal data structures for multiple sequences provided as input.

The gsufsort algorithm facilitates the construction of a suffix array for a collection of strings through the concatenation of these strings. Considering a collection of m strings, $s^1, s^2, \cdots, s^m$, derived from an alphabet $\Sigma = [1, \sigma]$ comprising ASCII symbols with respective lengths $n_1, n_2, \cdots, n_m$. The concatenation of these symbols results in a string $S = s^1\$s^2\$\cdots\$s^m\$\#$, where \$ represents the separator between distinct sequences, and # serves as the end-marker for the sequence. It is important to note that # and \$ do not appear in any string s^i, and $\# < \$ < \alpha$ for any other symbol $\alpha \in \Sigma$. The total length of S is given by $\sum_{i=1}^{m}(n_i + 1) + 1 = N$.

Before delving further into the intricacies of gsufsort, we take a moment to revisit the definitions of some data structures. Given a string S with a length of N, the suffix initiating at position i is denoted as S_i, where $0 \leq i < N$. The suffix array (SA) of a string S with a length of N comprises an array containing a permutation of $[0, N-1]$ that presents the suffixes of S in lexicographic order. The length of the longest common prefix shared by strings R and T is represented by $\text{lcp}(R,T)$. The LCP array for S supplies the lcp between consecutive suffixes in the order of SA, such that $\text{LCP}[0] = 0$ and $\text{LCP}[i] = \text{lcp}\left(S_{SA[i]}, S_{SA[i-1]}\right)$, where $0 < i \leq N-1$.

The gsufsort algorithm employs the gSACA-K method [Louza et al., 2017] to construct the SA for the concatenated string S. In doing so, it resolves ties between equivalent suffixes originating from distinct strings s^i and s^j based on their respective ranks, denoted by i and j. Notably, the gSACA-K algorithm is capable of concurrently computing the LCP and DA during the SA construction process, ensuring that LCP values do not surpass the separator symbols. The gSACA-K algorithm operates in $O(N)$ time complexity, utilizing $O(\sigma)$ working space.

2 LCP inervals Finding

The LCP-interval [Abouelhoda et al., 2002], or Longest Common Prefix interval, is a fundamental construct in the analysis of strings and their comparative properties. Formally, an LCP-interval is an Interval$[i..j]$ of lcp-value l_{min} in the LCP array, which satisfies the following properties:

1. $\text{LCP}[i] < l_{min}$,

2. $\text{LCP}[k] \geq l_{min}$ for all k with $i+1 \leq k \leq j$,
3. $\text{LCP}[k] = l_{min}$ for ai least one k with $i+1 \leq k \leq j$,
4. $\text{LCP}[j+1] < l_{min}$.

Algorithm S1 Compute LCP-intervals

Require: LCP array, minimum length threshold l_{min}

Ensure: LCP-intervals *intervals*

```
left ← 0, right ← 0, found ← false
while right < |LCP| do
    if LCP[right] ≥ l_min then
        if LCP[right] = l_min then
            found ← true
        end if
    else
        if found and right − left > 2 then
            push < left, right − 1 > into intervals
        end if
        left ← right
        found ← false
    end if
    right ← right + 1
end while
if found and right − left > 2 then
    push < left, right − 1 > into intervals
end if
```

An LCP-interval, as defined, encapsulates a contiguous subarray of the LCP array that adheres to specific conditions concerning the LCP values at the interval's boundaries and the elements within the interval. Specifically, within the LCP-interval, all elements possess LCP values that are greater than or equal to the lcp-value l_{min}. Moreover, the LCP values immediately preceding ($\text{LCP}[i]$) and succeeding ($\text{LCP}[j+1]$) the interval are strictly less than l_{min}. Consequently, an LCP-interval represents a region in the LCP array where the elements consistently exhibit LCP values that surpass those at the boundaries, which are comparatively smaller.

The process of identifying LCP-intervals, which represent contiguous sections of the LCP array that satisfy certain conditions, is a critical aspect of string analysis. Algorithm S1 provides an efficient method for extracting these intervals from a given LCP array.

The algorithm operates in a linear pass over the LCP array, leading to a time complexity of $O(n)$, where n is the size of the LCP array. The space complexity is also $O(n)$, as in the worst-case scenario, every position in the array could form a valid interval. This efficient design makes the algorithm highly practical for processing even large-scale data sets.

3 Extending LCP-intervals to MEMs

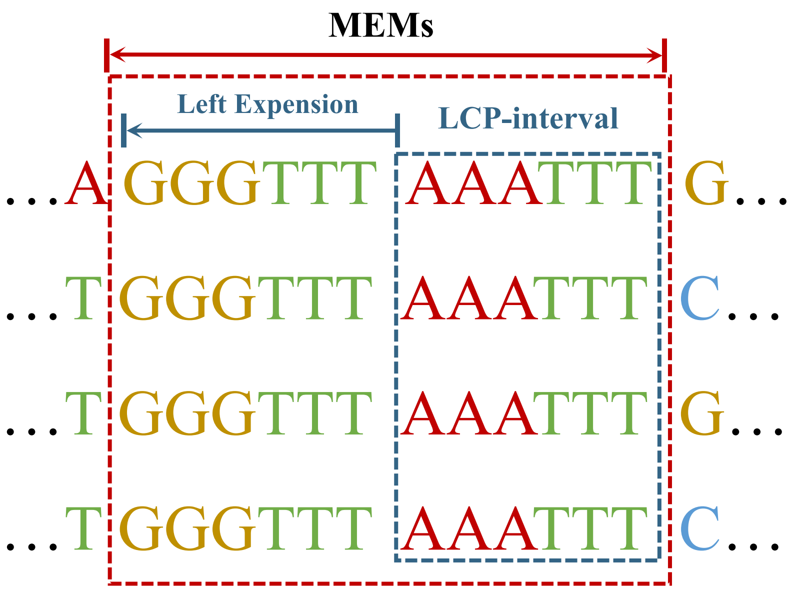

Figure S1: The method for extending LCP-intervals towards the left to identify a broader set of potential MEMs. Initially, each substring within an LCP-interval is inspected, focusing on the characters to its immediate left. If these characters are uniform, the interval extends leftwards by reducing the substring's start position and increasing its length. The process iteratively repeats until characters are no longer identical or the string's left boundary is reached, leading to the transformation of extended LCP-intervals into MEMs.

After identifying the LCP-intervals, our subsequent step is to explore their possibilities by extending towards the left, aiming to pinpoint a broader set of potential MEMs. By design, the LCP-intervals are bound such that they can't extend any further to the right. In contrast, leftward expansion is unrestricted and forms a crucial part of our comprehensive string data analysis. The leftward expansion is conducted in the following way. As shown in Fig S1, for each LCP-interval, we look at each substring it contains. We then inspect the characters immediately left of each substring. If all these characters are the same, we extend the LCP-interval to the left: the beginning position of each substring in the interval is reduced, and the length of each substring is increased. This process repeats iteratively, inspecting the newly included characters and further extending the interval to the left if they are all identical. The extension halts when we reach a stage where the characters left of the substrings are no longer identical or when we hit the left boundary of the original string, past which further extension is not possible. Through this detailed process, we extend the LCP-intervals to include a larger set of substrings, transforming them into MEMs.

4 MEMs Filtering

Although FMAlign2 employs MEMs and MUMmer [Marçais et al., 2018] uses MUM to segment sequences in a manner that seems quite similar, segmenting multiple sequences is a more complex problem than segmenting just two sequences. Considering the added challenge of segmenting multiple sequences, it's vital to emphasize the role of MEMs colinearity and size. If two MEMs each contain at most one substring per sequence and these substrings consistently maintain the same relative order across all sequences without overlap, We refer to these two MEMs as being colinear. Two MEMs being colinear means they won't conflict when segmenting sequences. Let's define the size of the i-th

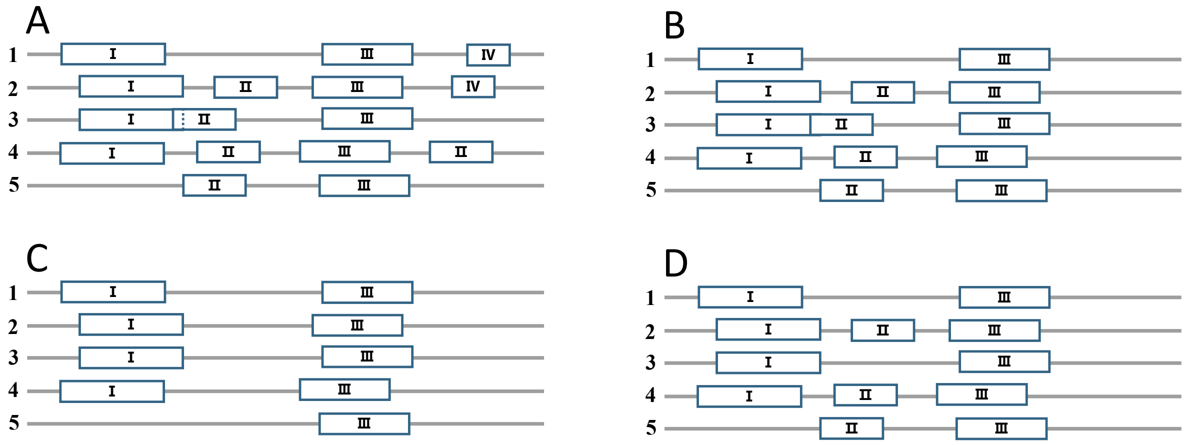

Figure S2: Four states of MEMs in filtering. (A) MEMs before preprocessing. (B) MEMs after preprocessing. (C) MEMs after global mode. (D) MEMs after local mode.

MEM as the product of its substring length l_i and the number of its substrings k_i:

$$size_i = l_i \times k_i \tag{1}$$

Given that larger MEMs are more likely to contribute to a MSA, our goal is to select a set of MEMs that not only have the largest size but are also colinear with each other. We will use Figure S2 to provide a specific example to illustrate the steps of MEMs filtering.

4.1 MEMs preprocessing

First, we perform a two-step preprocessing on each MEMs.

(1) We compute the average position of substrings within their respective sequences. For sequences with multiple substrings, we retain only the substring closest to this average position, guaranteeing that each MEMs contains at most one substring per sequence. For instance, in Figure S2A, the fourth sequence has two substrings under MEMs II. In Figure S2B, the substring that is farther from the average position is removed.

(2) We establish a minimum sequence coverage threshold $c (0 \leq c \leq 1)$. MEMs that do not cover a proportion greater than c of the total sequences n will be discarded. Subsequently, Given m retained MEMs, we sort them from left to right based on the average position of their substrings. In our example, the value of c is set to 0.6, meaning each MEMs must span across three sequences. Therefore, since MEMs IV spans only two sequences, it is entirely removed in Figure S2B.

4.2 Global and Local Mode

Algorithm S2 DP Algorithm of Global Mode

Require: $dp[0,\dots,n] = 0$ and $prev[0,\dots,n] = -1$, with n as $|MEMs|$
Ensure: $NewMEMs$ containing colinear MEMs with largest size

for each $i \in [0, n)$ **do**
  $size \leftarrow dp[i] + \text{MEMs}[i].\text{length} \times |\text{MEMs}[i]|$
  $dp[i] \leftarrow dp[i] + size$
  for each $j \in [0, i)$ **do**
    if $dp[j] + size > dp[i]$ and MEMs[i] and MEMs[j] are colinear **then**
      $dp[i], \text{prev}[i] \leftarrow dp[j], j$
    end if
  end for
end for
MaxSize $\leftarrow \max(dp)$
EndIndex $\leftarrow$ index of MaxSize in dp
Initialize $NewMEMs$ as an empty list
while EndIndex > 0 **do**
  Append EndIndex to $NewMEMs$
  EndIndex $\leftarrow$ prev[EndIndex]
end while
return $NewMEMs$

After preprocessing, we introduced two dynamic programming strategies to filter MEMs: the global mode, which focus on the entire MEMs, and the local mode, which concentrates on substrings within an individual sequence.

$$dp[i] = \begin{cases} \max\limits_{j<i, A[i,j]=1} (dp[j] + size_i) & \text{if } \exists j, A[i,j] = 1 \\ size_i & \text{otherwise} \end{cases} \tag{2}$$

Global Mode - In the global mode, we precompute a MEMs adjacency matrix $A \in \mathbb{R}^{m\times m}$ by examining the positions of substrings in two MEMs. If $A[i,j] = 1$, it signifies that the i-th MEMs and the j-th MEMs are colinear; otherwise, its value is 0. Based on the recursive formula 2, we could get the set of MEMs with the largest size. Therefore, as shown in Figure S2C, when there is an overlap between MEMs I and MEMs II, since MEMs I has a larger size, MEMs II is entirely removed. After verifying colinearity, we obtain the colinear MEMs set with the largest size as final result. The detailed explanation of the global mode is provided in the Algorithm 2.

$$dp[i] = \begin{cases} \max\limits_{j<i, A[i,j]=1} (dp[j] + l_i) & \text{if } \exists j, A[i,j] = 1 \\ l_i & \text{otherwise} \end{cases} \tag{3}$$

Local Mode - The global mode is straightforward, removing entire MEMs when faced with non-colinear instances. In contrast, the local mode, considering the permissible deletion of some MEM substrings, employs dynamic programming on all substrings of a sequence. For a given sequence with s MEMs substrings, these are sequentially numbered based on their start positions. We then construct a matrix $A \in \mathbb{R}^{s\times s}$ to discern overlaps; an entry $A[i,j] = 1$ implies non-overlap between the i-th and j-th substrings, and 0 indicates overlap. Using the recursive formula 3, we can obtain the set of non-overlapping substrings on this sequence with the maximum combined length. After executing the dynamic programming n times, all substrings not selected will be removed. MEMs with a substring count falling short of the floor value of $c \times n$ are eliminated, and the remaining MEMs are sorted based on average positions. As shown in Figure S2D, the dynamic programming on the third sequence will remove the substring of MEMs II, but the remaining number of substrings in MEMs II still meets the minimum sequence coverage and is thus retained. Ensuring substrings on each sequence align with the MEMs' order, we obtain the colinear MEMs set with the largest size.

5 Sequence-Profile Alignment

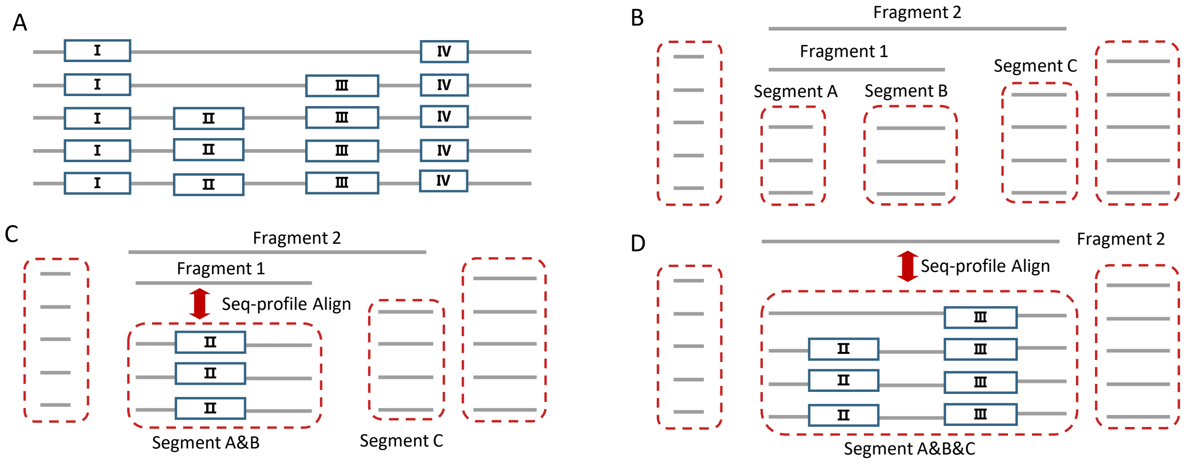

Figure S3: Sequence-Profile Alignment Example: (A) In this example, there are 4 partial chains, with the second and third chains not spanning the entire sequence. (B) After cutting the sequence by partial chains, a total of 5 segments and two fragments are generated. The three segments that will be involved subsequently are marked as A, B, C. Each Segment will undergo multiple sequence alignment, which is not shown in the figure. (C) The sequences with fragments are the first and second ones, and since the fragment on the second sequence is shorter than that on the first, we prioritize aligning fragment 1 from the second sequence to the backbone. The creation of fragment 1 is due to the cutting by partial chains 1 and 3; hence, we first splice segments A and B as well as partial 2 to form Segment A&B. Fragment 2 and Segment A&B are then aligned using sequence-profile alignment. (D) Fragment 1 is aligned to the backbone using the same steps, with this backbone being formed by the previous alignment results, partial chain 3, and segment C.

6 Refinement

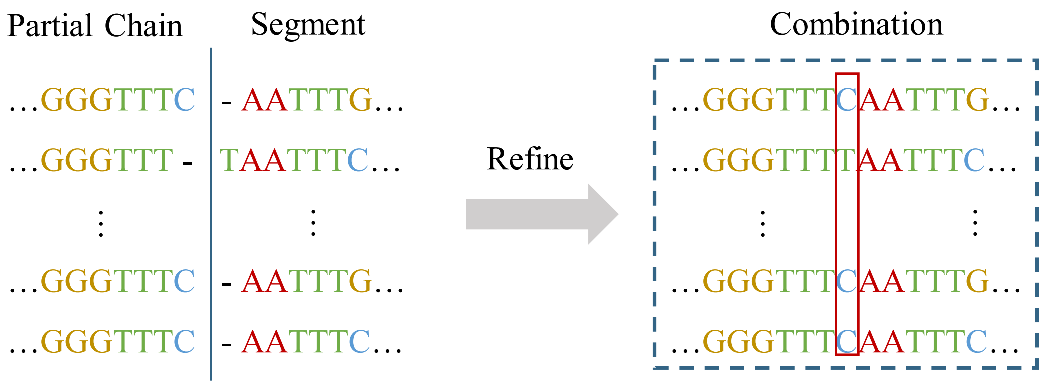

Figure S4: Refinement case: The partial chain's rightmost column mainly shows 'C', except for a gap at the second spot. The segment's leftmost column mostly has gaps, but a 'T' at the second position. The refinement seamlessly merges these two columns into the unified sequence highlighted in the red box on the right.

7 Comparison of eight methods on simulated different length datasets

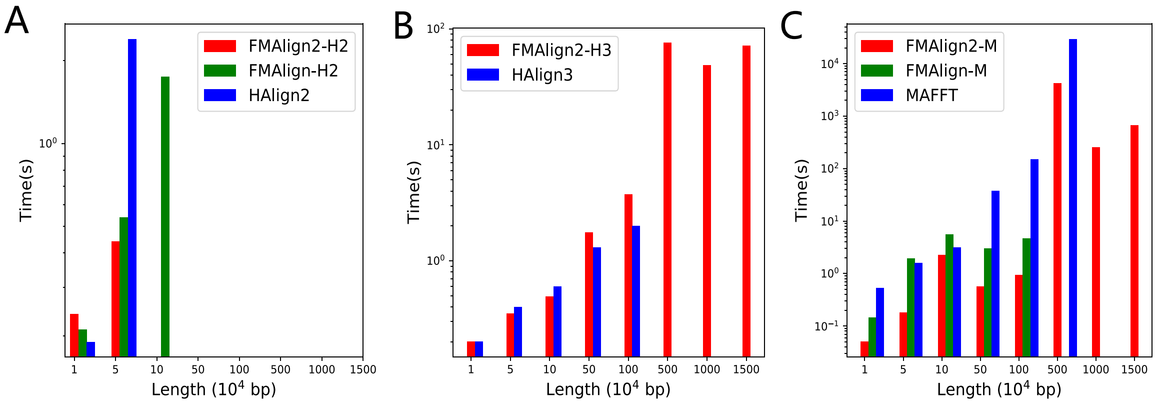

Figure S5: Comparison of eight methods under different length datasets. The absence of the methods without bar charts is due to memory overflow, except for MAFFT, which exceeded the acceptable range of alignment time. (A) Comparison of methods with HAlign2 as the core under different length datasets. (B) Comparison of methods with HAlign3 as the core under different length datasets. (C) Comparison of methods with MAFFT as the core under different length datasets.

8 Comparison of eight methods under real datasets

Table S1: Detailed information of dataset

Datasets	N	Length			Size
		MIN	MAX	AVG	
mt (1x)	672	16 556	16 579	16 569	10MB
mt (20x)	13 440	16 556	16 579	16 569	222.9MB
mt (50x)	33 600	16 556	16 579	16 569	557.3MB
mt (100x)	66 000	16 556	16 579	16 569	1.1 GB
VARV	4	186 108	186 677	186 394	0.74MB
M. genitalium	4	579 504	579 977	579 709	2.24MB
M. bovis	4	969 233	990 850	980 464	3.79MB
S. pneumonia	4	2 111 882	2 184 682	2 160 522	8.35MB
E. coli	4	4 578 159	4 686 137	4 633 446	17.9MB

Table S2: Time consumption and average SP scores for mitochondrial genome database

Method	mt (1x)		mt (20x)		mt (50x)		mt (100x)	
	Time	Average SP	Time	Average SP	Time	Average SP	Time	Average SP
MAFFT	13.98s	159.83	17m35s	156.4	1h2m36s	156.41	2h44m21s	158.3
HAlign2	17.69s	152.3	4m50s	184.26	12m6s	184.26	23m20s	184.26
HAlign3	1.77s	152.3	9.83s	152.24	19.38s	152.24	39.13s	152.24
FMAlign-M	8.19s	154.44	10m26s	154.35	41m29s	154.36	2h44m40s	156.36
FMAlign-H2	7.65s	159.02	3m31s	158.95	11m12s	158.95	19m38s	158.95
FMAlign2-M	5.46s	150.7	5m32s	152.52	35m56s	152.47	1h11m52s	152.26
FMAlign2-H2	3.62s	184.32	2m3s	184.26	5m18s	184.26	10m44s	184.26
FMAlign2-H3	2.84s	152.39	1m50s	152.33	4m40s	152.33	9m32s	152.33

Table S3: Peak memory usage for mitochondrial genome database

Method	mt (1x)	mt (20x)	mt (50x)	mt (100x)
MAFFT	5.99GB	4.01GB	10.68GB	22.05GB
HAlign2	4.68GB	8.41GB	12.07GB	21.02GB
HAlign3	245.01MB	7.04GB	13.82GB	22.85GB
FMAlign-M	381.84MB	12.05GB	59.77GB	215.23GB
FMAlign-H2	2.50GB	25.39GB	35.46GB	51.01GB
FMAlign2-M	931.78MB	32.21GB	38.18GB	94.60GB
FMAlign2-H2	3.93GB	20.41GB	40.59GB	97.56GB
FMAlign2-H3	2.95GB	21.56GB	48.81GB	92.11GB

Table S4: Peak memory usage for virus genome datasets

Method	*VARV*	*M. genitalium*	*M. bovis*	*S. pneumonia*	*E. coli*
MAFFT	116.33MB	375.19MB	734.21MB	533.27MB	1.11GB
HAlign2	-	-	-	-	-
HAlign3	112.27MB	124.82MB	136.54MB	-	-
FMAlign-M	39.43MB	132.62MB	239.17MB	6.56GB	6.67GB
FMAlign-H2	664.82MB	644.34MB	1.31GB	-	-
FMAlign2-M	9.93MB	2.03GB	177.23MB	13.13GB	4.01GB
FMAlign2-H2	70.08MB	3.71GB	8.98GB	66.34GB	53.24GB
FMAlign2-H3	991.45MB	1.73GB	2.17GB	6.88GB	33.29GB

Note: '-' indicates that the method cannot complete the alignment on the dataset due to memory overflow.

9 Sortware Parameter

Linux user: `./FMAlign2 -i /path/to/data [other options]`

Windows user: `./FMAlign2.exe -i /path/to/data [other options]`

Show parameters details: `./FMAlign2 -h`

Parameters Details:

-i ***[file path]*** [required] The path to the input file.

-o ***[output path]*** [default: ouput.fmaligned2.fasta] The path to the output file.

-p ***[package]*** [default: mafft] The MSA method used in parallel align. Examples include halign3, halign2, and mafft.

-t ***[int]*** [default: cpu number] The maximum number of threads that the program can run. It is recommended to set this to the number of CPUs.

-l ***[int]*** [default: square root of mean length] The minimum length of MEMs, the default value is the square root of the mean length of the sequences.

-c ***[float]*** [default: 1] A floating-point parameter that specifies the minimum coverage across all sequences. Acceptable values range from 0 to 1.

-f ***[mode]*** [default: global or local] The filter MEMs mode. If the sequence number is less than 100, the local mode is used; otherwise, the global mode is used.

-d ***[int]*** [default:0] The depth of recursion, which can generally be ignored.

-v ***[int]*** [default:1] The verbose option, can be set to 0 or 1. This can usually be ignored.

-h ***[help]*** To print help information.

References

M. I. Abouelhoda, S. Kurtz, and E. Ohlebusch. The enhanced suffix array and its applications to genome analysis. In *Algorithms in Bioinformatics: Second International Workshop, WABI 2002 Rome, Italy, September 17–21, 2002 Proceedings 2*, pages 449–463. Springer, 2002.

F. A. Louza, S. Gog, and G. P. Telles. Inducing enhanced suffix arrays for string collections. *Theoretical Computer Science*, 678:22–39, 2017.

F. A. Louza, G. P. Telles, S. Gog, N. Prezza, and G. Rosone. gsufsort: constructing suffix arrays, lcp arrays and bwts for string collections. *Algorithms for Molecular Biology*, 15:1–5, 2020.

U. Manber and G. Myers. Suffix arrays: a new method for on-line string searches. *siam Journal on Computing*, 22(5):935–948, 1993.

G. Marçais, A. L. Delcher, A. M. Phillippy, R. Coston, S. L. Salzberg, and A. Zimin. Mummer4: A fast and versatile genome alignment system. *PLoS computational biology*, 14(1):e1005944, 2018.

S. Muthukrishnan. Efficient algorithms for document retrieval problems. In *SODA*, volume 2, pages 657–666. Citeseer, 2002.
